# Functional testing of topical skin formulations using an optimised ex vivo skin organ culture model

**DOI:** 10.1007/s00403-016-1645-8

**Published:** 2016-04-16

**Authors:** G. P. Sidgwick, D. McGeorge, A. Bayat

**Affiliations:** Plastic and Reconstructive Surgery Research, Faculty of Medicine and Human Sciences, Manchester Academic Health Science Centre, Institute of Inflammation and Repair, University of Manchester, Stopford Building, Manchester, M13 9PT UK; Centre for Dermatology Research, Institute of Inflammation and Repair, University of Manchester, Manchester, UK; Grosvenor Nuffield Hospital, Wrexham Road, Chester, UK; Healthcare Science Research Centre, Manchester Metropolitan University, Manchester, UK

**Keywords:** Skin scarring, Dermal delivery, Extracellular matrix, Mast cells, Inflammation, Dermatology

## Abstract

A number of equivalent-skin models are available for investigation of the ex vivo effect of topical application of drugs and cosmaceuticals onto skin, however many have their drawbacks. With the March 2013 ban on animal models for cosmetic testing of products or ingredients for sale in the EU, their utility for testing toxicity and effect on skin becomes more relevant. The aim of this study was to demonstrate proof of principle that altered expression of key gene and protein markers could be quantified in an optimised whole tissue biopsy culture model. Topical formulations containing green tea catechins (GTC) were investigated in a skin biopsy culture model (*n* = 11). Punch biopsies were harvested at 3, 7 and 10 days, and analysed using qRT-PCR, histology and HPLC to determine gene and protein expression, and transdermal delivery of compounds of interest. Reduced gene expression of α-SMA, fibronectin, mast cell tryptase, mast cell chymase, TGF-β1, CTGF and PAI-1 was observed after 7 and 10 days compared with treated controls (*p* < 0.05). Histological analysis indicated a reduction in mast cell tryptase and chymase positive cell numbers in treated biopsies compared with untreated controls at day 7 and day 10 (*p* < 0.05). Determination of transdermal uptake indicated that GTCs were detected in the biopsies. This model could be adapted to study a range of different topical formulations in both normal and diseased skin, negating the requirement for animal models in this context, prior to study in a clinical trial environment.

## Introduction

A range of different models exist for studying the effect of drugs and cosmaceuticals, both in vitro and in vivo. However, studying the effect of drug and cosmaceutical compounds can be challenging. In March 2013, the use of animal models for cosmetic testing of products or ingredients for sale in the EU was banned [32]. Animal models were not perfect; mice, rat and rabbit models in particular are limited as they do not accurately represent the structure of human skin, the best current model in terms of dermal structure and underlying mechanisms is the pig [16, 35, 44]. The field of dermocosmetics and our increased knowledge about the architecture of skin has led to an enhancement and refinement of a number of in vitro, in vivo and in silico techniques to aid skin research, [9, 11] which can be adapted to study a range of conditions in order to more accurately study both human skin disease [34] and aid drug development [28].

Whilst in vitro models have their benefits, they are not without their drawbacks. Two dimensional cell culture models do not represent the interactions and mechanisms present in whole skin, such as the effect of the extracellular matrix (ECM) in terms of structure, as well as cell signalling mechanisms and the processes of metabolism [18]. The incorporation of skin appendages such as hair follicles and sweat glands is an added complication [28]. It is also more challenging to model the effects of dermal absorption; while keratinocytes in air exposed culture can differentiate and form layers and produce a stratum corneum, this is not representative of normal skin structure [22]. Ex vivo human skin tissue is more appropriate for certain types of research; use of whole skin biopsies in culture allows the effect of individual ingredients and formulations to be tested in an environment more closely mimicking normal skin [33].

In this study, five unique cream formulations and controls containing different quantities and proportions of green tea catechins (GTC) were assessed, in order to validate and optimise a normal skin tissue biopsy organ culture model already established in our laboratory [12, 1, [27]. The effect of these formulations at the gene and protein expression level was assessed, as well as mast cell numbers, which are linked with inflammation and increased in a range of dermatological conditions [3, 17, 23, 26, 30, 39]. The level of transdermal green tea extract delivery was also analysed using a previously published method [2], to interpret in vitro effect in a whole tissue biopsy culture model in normal skin.

## Materials and methods

### Tissue samples

Normal skin tissue samples (*n* = 11) were obtained by the Skin and Tissue Bank (North West Research Ethics Committee Ref 11/NW/0683). Patients were recruited and samples obtained following informed consent prior to elective cosmetic surgery, and were anonymised and coded prior to use. Samples were stored and transported at 4 °C in Dulbecco’s Modified Eagle Medium (DMEM) and processed for whole tissue organ culture within 24 h of excision.

### Organ culture model

**Fig. 1:**
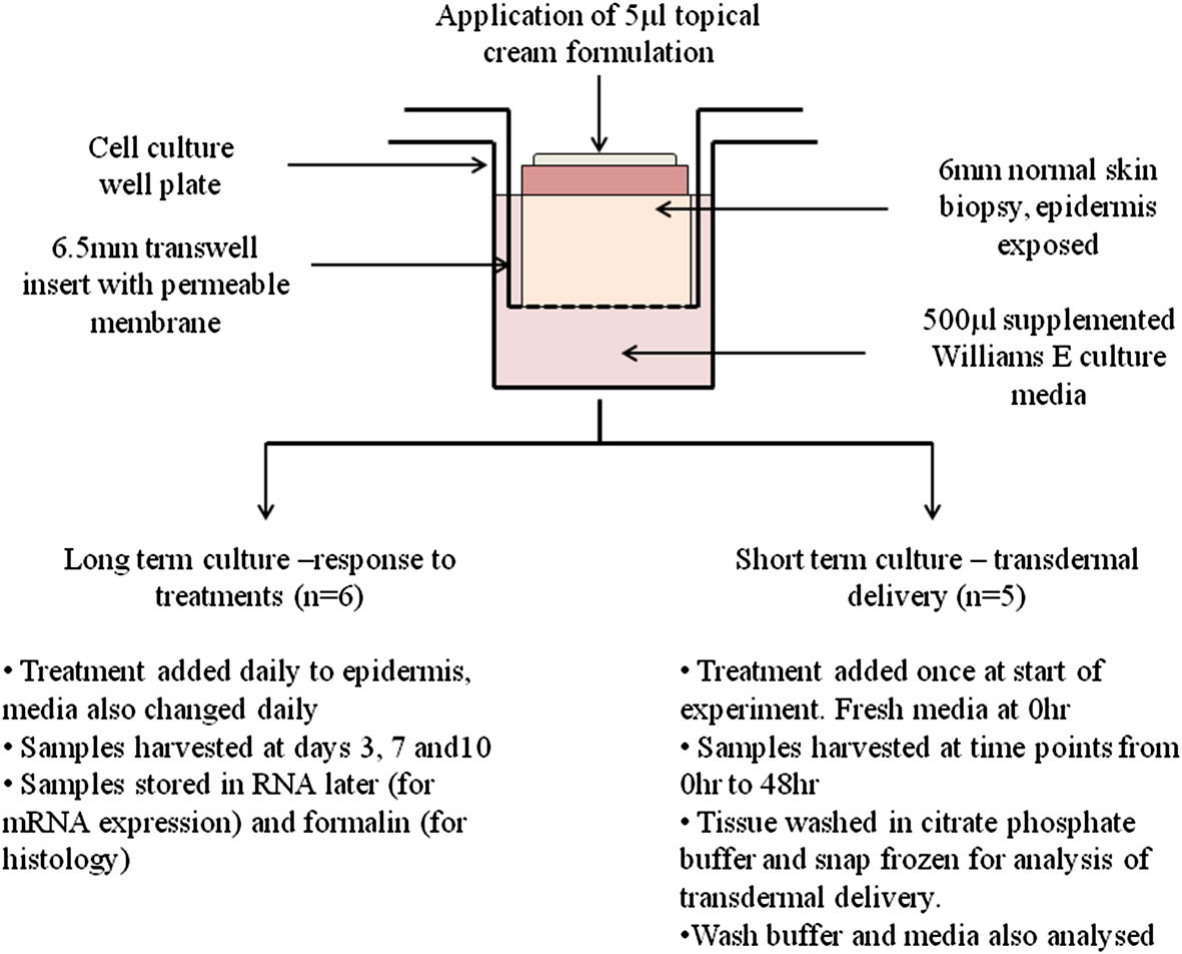
A diagram and flow chart depicting the organ culture methodology, and the experimental approach used in this study

The normal skin organ culture was based on the methodology of Lu et al. [27]. Six millimetre punch biopsies of normal skin were added to a 24-well plate containing transwell inserts, allowing the biopsy to be suspended in liquid culture media whist keeping the epidermis separate and exposed to air for the addition of topical treatments (Fig. 1). Supplemented serum free Williams E culture media was used (1 % penicillin/streptomycin, 1 % l-glutamine, 1 % non-essential amino acid solution, 1 % ITS + 3, and 10 ng/ml hydrocortisone), 500 µl of media was added to each well and was changed daily. Antibiotics were used in order to prevent contamination by the microflora naturally present on human skin. The culture model was maintained in an incubator under standard conditions (37 °C, 5 % CO_2_).

For long term culture assessment, 5 µl of each topical formulation was added daily to the epidermal region of each punch biopsy (*n* = 6). Five different cream formulations and controls were used (Table 1). Eucerin cream containing 10 % Urea (Eucerin, Germany) was used as a control cream; this is a common over the counter moisturiser used across a range of dermatological conditions to reduce itch and inflammation. The organ culture was maintained for 3, 7 or 10 days, upon which biopsies were removed and washed with PBS to remove any remaining cream from the stratum corneum. Biopsies were either fixed in formalin and embedded in wax for histological analysis, or stored in RNA later at −80 °C prior to mRNA extraction and quantification.

**Table 1:** List of the active ingredients included in the various treatments

Cream name	Active ingredients
A—cream formulation 1: green tea extract	Aqua, Dipropylene Glycol Dipelargonate, Caprylic/Capric Triglyceride, Glycerin, Cyclopentasiloxane, Cetyl Alcohol, Cetearyl Alcohol, Phenoxyethanol, Glyceryl Stearate, Peg-75 Stearate, Ceteth-20, Steareth-20, Poloxamer 235, Poloxamer 338, Ethoxydiglycol, Xanthan Gum, **Camellia Sinensis Extract**, Butylene Glycol, Tocophersolan, **Tocopherol**, Maltodextrin, Triethanolamine, Acrylates/C10-30 Alkyl Acrylate Crosspolymer, Ethylhexylglycerin, Helianthus Annuus Seed Oil, Lecithin, **Hydrolyzed Algin**, Maris Aqua, **Chlorella Vulgaris Extract, Magnolia Officinalis Bark Extract**, **Vitis Vinifera Seed Extract**
B—base cream formulation 1: (control)	Aqua, Dipropylene Glycol Dipelargonate, Caprylic/Capric Triglyceride, Glycerin, Cyclopentasiloxane, Cetyl Alcohol, Cetearyl Alcohol, Phenoxyethanol, Glyceryl Stearate, Peg-75 Stearate, Ceteth-20, Steareth-20, Poloxamer 235, Poloxamer 338, Ethoxydiglycol, Xanthan Gum, Butylene Glycol, Tocophersolan, Tocopherol, Maltodextrin, Triethanolamine, Acrylates/C10-30 Alkyl Acrylate Crosspolymer, Ethylhexylglycerin, Helianthus Annuus Seed Oil, Lecithin, **Hydrolyzed Algin,** Maris Aqua, **Chlorella Vulgaris Extract, Magnolia Officinalis Bark Extract**, **Vitis Vinifera Seed Extract**
C—cream formulation 2: green tea extract	Aqua, Glycerin, Olus (vegetable) oil, Rosa Canina Fruit Oil, Triticum Vulgare, (Wheat) Germ Oil, **Epigallocatechin Gallate**, Cera Alba, Glyceryl Stearate, Citrate, Cetearyl Alcohol, Glyceryl Caprylate, Benzyl Alcohol, Salicylic, Acid, Glycerin, Sorbic Acid, Allantoin, Xanthan Gum, Arginine, **Aloe Barbadensis Leaf Juice, Tocopherol, Sodium Hyaluronate**
D—base cream formulation 2: (control)	Aqua, Glycerin, Olus (vegetable) oil, Rosa Canina Fruit Oil, Triticum Vulgare, (Wheat) Germ Oil, Cera Alba, Glyceryl Stearate Citrate, Cetearyl, Alcohol, Glyceryl Caprylate, Benzyl Alcohol, Salicylic Acid, Glycerin, Sorbic Acid, Allantoin, Xanthan Gum, Arginine, **Aloe Barbadensis Leaf Juice, Tocopherol, Sodium Hyaluronate**
Cream E: Eucerin (10 % urea) control cream—commercially available moisturizer	Aqua, Benzyl Alcohol, Caprylic/Capric Triglyceride, Dimethicone, Glycerin, Hydrogenated Castor Oil, Isopropyl Palmitate, Lactic Acid, Magnesium, Sulfate, Methoxy PEG-22-Dodecyl Glycol Copolymer, Octyldodecanol, Ozokerite, Cera Microcristallina, PEG-2 Hydrogenated Castor Oil, PEG-45 Dodecyl Glycol, Copolymer, PEG-7 Hydrogenated Castor Oil, Sodium Lactate, Sorbitan, Isostearate, **Urea**
Untreated control	No topical treatment

Cream A and B were the same formulation, with cream A containing the active ingredients and cream B being the base cream control

Cream C and D were a different formulation, with cream C containing the active ingredient and cream D being the base cream control. Two further controls were included—Cream E was Eucerin (10 % Urea) moisturiser, and a further set of biopsies which were untreated. Active ingredients are indicated in bold

### Transdermal delivery quantification

A second set of short term culture experiments were performed, in order to assess of transdermal delivery, the normal skin organ culture model was set up as described (*n* = 5), and left to establish for 48 h (see also Fig. 1). One 5 µl of Cream A, containing GTC, was added to the epidermal region, and biopsies were removed at time points over a further 48 h period. The biopsies were washed in 500 µl of citrate phosphate buffer (18.1 g Na_2_HPO_4_·12H_2_O, 9.42 g citric acid, 1 ml TFA in 1 L de-ionised water, adjusted to pH 5.5 with concentrated NaOH), then snap frozen in liquid nitrogen and stored at −80 °C for analysis of uptake of components of GTE. The citrate phosphate wash buffer and culture media were also retained at each time point and stored at −80 °C for analysis.

### Analysis of gene expression

The expression of mRNA from organ culture punch biopsy samples was assessed. The mRNA was extracted using Qiagen Qiashredder and RNAeasy mini prep kits (as per manufacturer’s protocol) and samples were stored at −80 °C prior to use. The concentration of mRNA obtained was quantified using a nanodrop 2000 prior to conversion into cDNA. Samples were analysed using the Roche qRT-PCR Lightcycler 480 platform with RPL32 gene expression used for quantification and standardisation of results. A list of primers and sequences used is shown in Table 2.

**Table 2:** A list of PCR primers used in this study, along with the Roche probe ID

Primers	Sequence	Probe
Collagen I		
L	ctgtacgcaggtgattggtg	15
R	atgttcagctttgtggacctc	15
Fibronectin		
L	gccactggagtctttaccaca	64
R	cctcggtgttgtaaggtgga	64
α-SMA		
L	ctgttccagccatccttcat	58
R	tcatgatgctgttgtaggtggt	58
TGF-β1		
L	agtggttgagccgtggag	68
R	tgcagtgtgttatccctgct	68
MC Triptase		
L	gcgatgtggacaatgatgag	6
R	tccattatggggaccttcac	6
MC Chymase		
L	acggaactttgtgctgacg	4
R	ggctccaagggtgactgtta	4
CTGF		
L	ccgtactcccaaaatctcca	71
R	ttagctcggtatgtcttcatgc	71
PI3-K		
L	ttgactttgaggtagtccagacc	71
R	aaaagtgtccctgttgattcttct	71
PAI-1		
L	aaactccctagtctccacctga	14
R	ccttaagggagttgtgcttca	14
STAT3		
L	tgatgcagtttggaaataatgg	18
R	catgtcaaaggtgagggactc	18
MMP-3		
L	caaaacatatttctttgtagaggacaa	36
R	ttcagctatttgcttgggaaa	36
RPL32		
L	gaagttcctggtccacaacg	17
R	gagcgatctcggcacagta	17

### Histology

Slides were prepared from wax embedded whole tissue blocks using a Leica microtome and left to dry, with consecutive tissue sample in duplicate on each slide. Slides were dewaxed with xylene and ethanol and rehydrated. Antigen retrieval was performed using citrate buffer, pH 6, for 60 min in a water bath at 60 °C. Immunofluorescence slides were washed with PBS/0.01 % Tween 20 and blocked with 2 % BSA in PBS prior to addition of primary antibodies (Abcam, Cambridge UK). After incubation overnight at 4 °C, slides were washed with TBS buffer containing 0.05 % Triton X100 followed by addition of secondary antibody (Life Technologies, Paisley UK). Slides were counterstained with DAPI, mounted and stored at −20 °C prior to analysis. Immunohistological slides were prepared according to the Leica novolink peroxidise staining kit protocol (Milton Keynes, UK), with overnight incubation of primary antibody at 4 °C, and development of peroxidase staining with DAB chromogen reagent for 5 min. Slides were also prepared and stained with haematoxylin and eosin in order to assess morphological and histological changes in the punch biopsies in culture over time. All tissue sections were stained and prepared in the same batch in order to reduce variability.

### HPLC determination of green tea derived compounds in the organ culture model

**Fig. 2:**
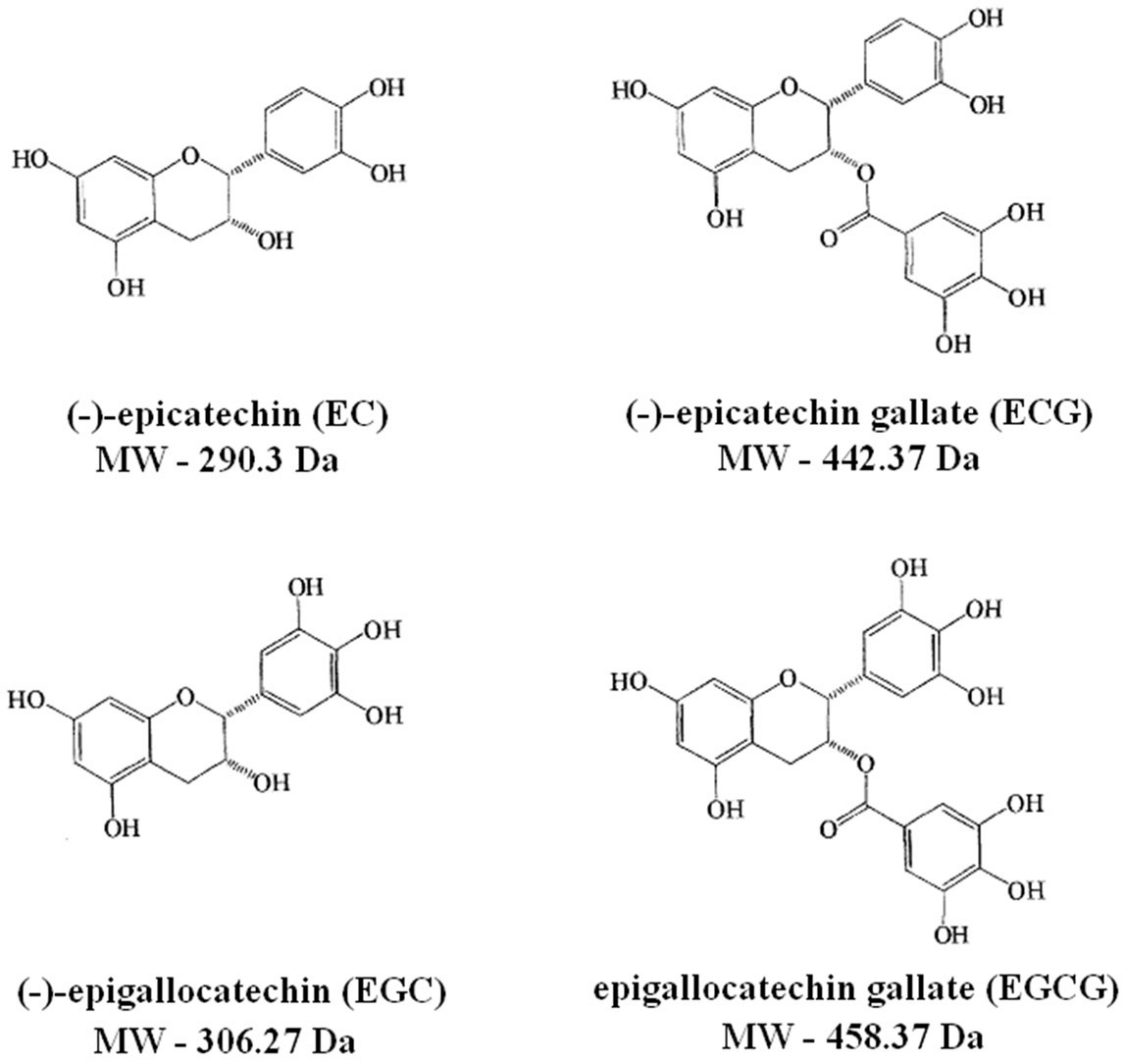
The chemical structures and molecular weights of the four main green tea catechins (GTCs), (-)-epicatechin (EC), (-)-epigallocatechin (EGC), (-)-epicatechin gallate (ECG) and (-)-epigallocatechin gallate (EGCG)

HPLC determination of uptake of GTC derived compounds was undertaken, based on the methodology of Batchelder et al. [2]. In brief, an Agilent 1100 system was utilised and data analysis undertaken using chemstation software. The column used was a Phenomenex Sphereclone (5 µm ODS, 4.6 × 250 mm), with a mobile phase of 0.1 % TFA:Acetonitrile in the ratio of 87:13. The flow rate was set at 1.0 ml/min with no gradient, 50 µl of each sample was run for 40 min and detection of the major catechins found in green tea, (-)-epicatechin (EC), (-)-epigallocatechin (EGC), (-)-epicatechin gallate (ECG) and (-)-epigallocatechin gallate (EGCG) was undertaken at 210 nm (Fig. 2). Calibration curves of pure catechins dissolved in DMSO were prepared, diluted in citrate phosphate buffer.

To test for transdermal uptake of green tea catechins, tissue samples stored at −80 °C were defrosted and separated finely minced with a scalpel. 500 µl of citrate phosphate buffer was added to each tissue fragment, which was then lysed in a tissue lyser for 2 min. After 60 min incubation, samples were centrifuged and the supernatant quantified on the HPLC. Culture media was also analysed to test for catechin concentrations, as was the wash buffer used to wash the tissue samples prior to storage.

### Statistics and data analysis

Analysis of PCR data was undertaken in Microsoft Excel, utilising the Paired Student’s *T* Test to define significance as *p* < 0.05. PCR data was expressed as fold change, via 2^−(ΔΔCT)^. Analysis of histological data was performed using Definiens Tissue Studio (Definiens AG, Munich, Germany), in order to quantify the amount of staining present. For each batch of microscope slides, whole sections of stained tissue in duplicate were scanned, with exposure settings standardised in order to eliminate variability.

## Results

### Analysis of gene expression

**Fig. 3:**
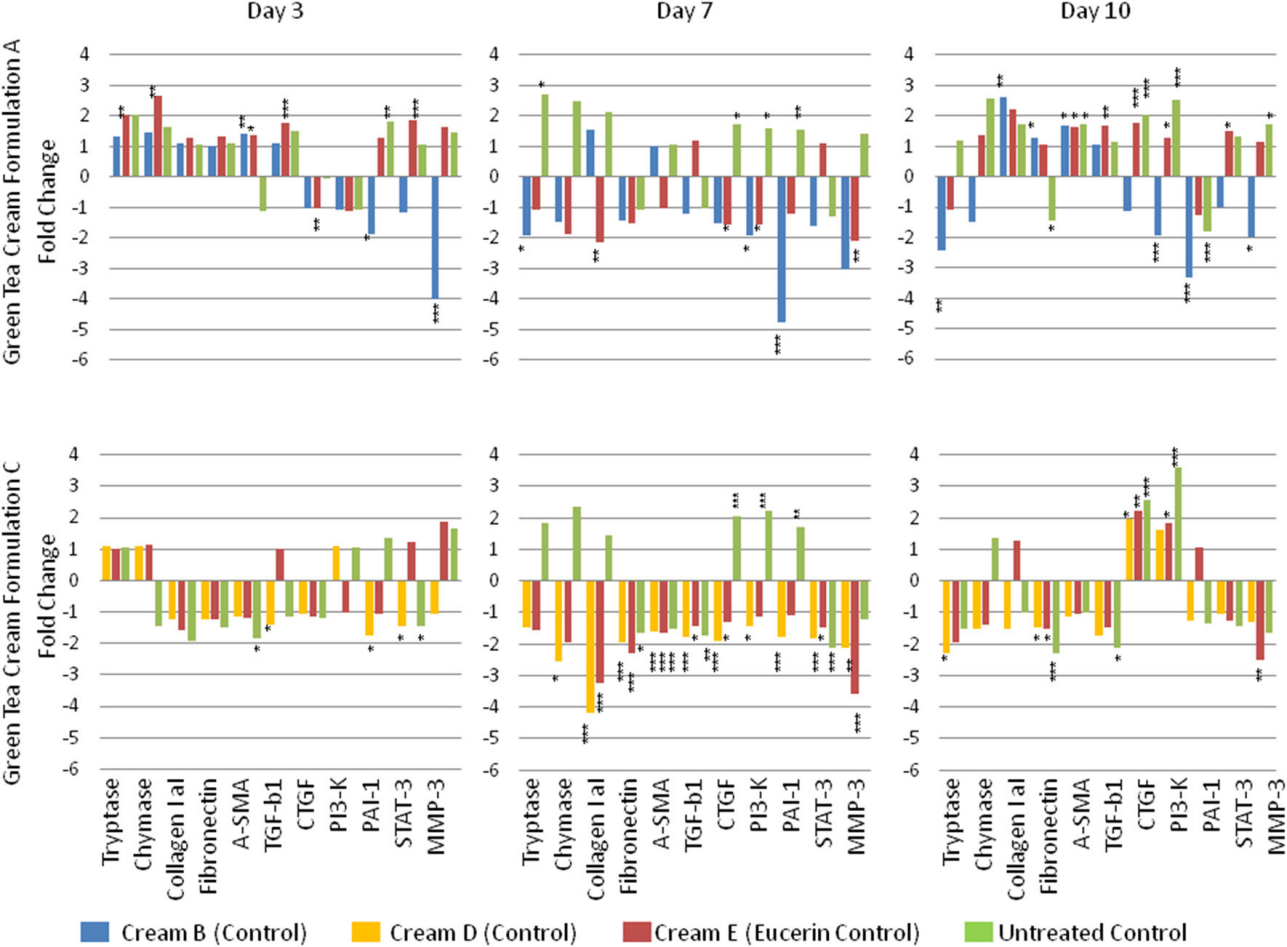
Comparison of fold change in gene expression in the organ culture models treated with cream A (*top row*) and cream *C* (bottom row) after 3, 7 and 10 days (First, second and third *columns*, respectively) compared with the treated and untreated controls, as indicated in the key. (Statistical significance as derived from the students *T* test indicated as *p < 0.05, **p < 0.01, ***p < 0.001)

In the long term organ culture experiments, gene expression was determined after 3, 7 and 10 days, treated daily with the five cream formulations (*n* = 6). An untreated biopsy taken at day 0 was used as a starting control, with the untreated biopsies and treated control biopsies taken from the organ culture model used as internal controls. mRNA expression between day 0 and later time points was reduced by approximately tenfold (*p* < 0.001), due to the fact that the tissue biopsies in culture are no longer actively proliferating. The PCR data comparing cream A and cream C were broadly similar (Fig. 3), however there were some differences, in particular with collagen I, fibronectin, TGF- β1 and α-SMA, related to the additional active components in the cream A formulation.

For cream A, at Day 3 mast cell tryptase (*p* < 0.01), mast cell chymase (*p* < 0.01), TGF-β1 (*p* < 0.001) and STAT-3 (*p* < 0.001) were increased compared with cream E control (Eucerin), whereas CTGF was reduced (*p* < 0.01). PAI-1 (*p* < 0.05) and MMP-3 (*p* < 0.001) were reduced compared with cream B control, but PAI-1 was increased compared with untreated control (*p* < 0.01). α-SMA was increased compared to cream B (*p* < 0.01) and Eucerin (*p* < 0.05) controls.

At Day 7, mast cell tryptase was increased compared with untreated control (*p* < 0.05), but reduced compared with cream B (control) (*p* < 0.05). PI3-K (*p* < 0.05), CTGF (*p* < 0.05) and MMP-3 (*p* < 0.01) were reduced compared to cream E control. PAI-1 (*p* < 0.001) and P13-k (*p* < 0.05) were reduced compared with cream B control. CTGF (*p* < 0.05), PAI-1 (*p* < 0.01) and PI3-K (*p* < 0.05) were increased compared to untreated control.

At Day 10, mast cell tryptase (*p* < 0.01), PI3-K (*p* < 0.001), PAI-1 (*p* < 0.001), and MMP-3 (*p* < 0.05) were reduced compared with cream B (control), whereas fibronectin was increased (*p* < 0.01). Fibronectin was reduced compared to untreated control (*p* < 0.01) whereas PA3-K was increased (*p* < 0.001). Collagen 1 expression was increased compared with cream B (*p* < 0.01). CTGF (*p* < 0.001), MMP-3 (*p* < 0.05) and PI3-K (*p* < 0.001) were increased compared with untreated controls, and TGF-β1 (*p* < 0.01), CTGF (*p* < 0.001), STAT-3 (*p* < 0.05) and PI3-K (*p* < 0.05) were increased compared with cream E control. α-SMA was increased compared to all controls (*p* < 0.01).

For cream C, at Day 3 α-SMA and STAT-3 was reduced compared to untreated control (*p* < 0.05), with TGF- β1 PAI-1 and STAT-3 reduced compared with cream D (*p* < 0.05). At Day 7 mast cell chymase (*p* < 0.05), collagen I (*p* < 0.001), CTGF (*p* < 0.001), PI3-K (*p* < 0.001), PAI-1 (*p* < 0.001) and MMP-3 (*p* < 0.01) were reduced compared with cream D (control). collagen 1 (*p* < 0.001), CTGF (*p* < 0.05) and MMP-3 (*p* < 0.001) were also reduced compared with cream E control, with Fibronectin (*p* < 0.05), α-SMA (*p* < 0.001), TGF-β1 (*p* < 0.05) and STAT-3 (*p* < 0.05) reduced compared with all controls. CTGF (*p* < 0.001), PI3-K (*p* < 0.001) and PAI-1(*p* < 0.01) were increased compared to untreated control.

At Day 10, mast cell tryptase was reduced compared with cream D (control) (*p* < 0.05). Fibronectin was reduced compared with all controls (*p* < 0.05) whereas CTGF was increased (*p* < 0.05). TGF-β1 was reduced compared with untreated control (*p* < 0.05) and MMP-3 reduced compared with cream E control (*p* < 0.01). PI3-K was increased compared with untreated control (*p* < 0.001) and cream E control (*p* < 0.05).

### Detection of green tea catechin uptake

**Fig. 4:**
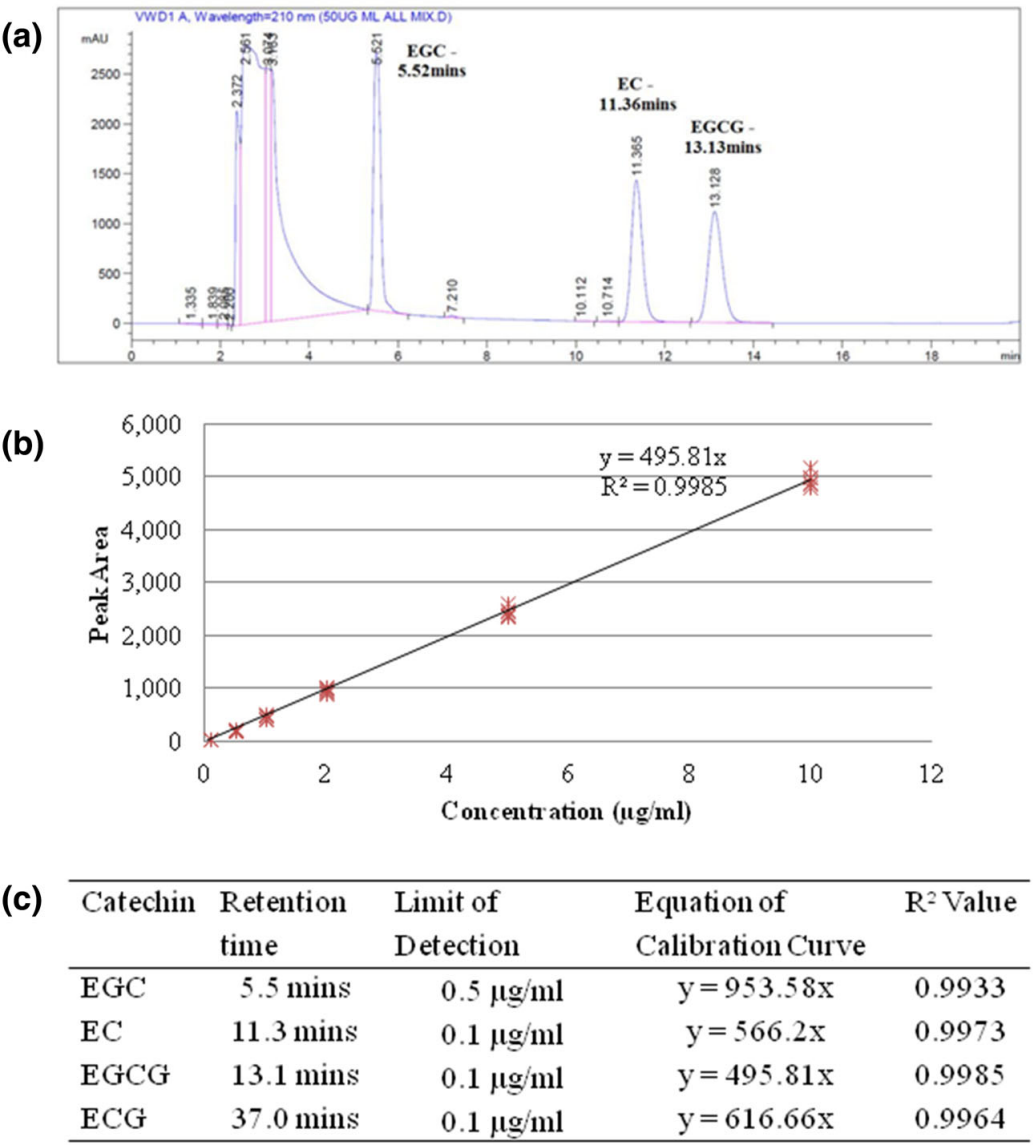
Details of the HPLC calibration experiments performed. **a** An example HPLC trace, indicating the resolution of the EGC (~5.5 min), EC (~11.3 min) and EGCG (~13.1 min) peaks observed in a mixed calibration curve sample. Pure GTC compounds were solubilised separately, and following serial dilution were injected at increasing concentrations. ECG peak at ~37 min is not shown due to scale. **b** An example calibration *curve* for EGCG (*n* = 6 separate calibration curves), the most commonly found and bioreactive GTC found in *C*. *sinensis*. **c** Characteristics of the individual GTC HPLC calibration curves (*n* = 6), indicating the reproducibility and linearity of the protocol and methodology

Peaks were observed for EGC, EC, EGCG and ECG at retention times of 5.5, 11.3, 13.1 and 37.0 min respectively, when pure compound was dissolved in DMSO and then diluted with citrate phosphate buffer. Each peak was individually resolved (Fig. 4a, ECG peak at 37 min not shown due to scale). A full EGCG calibration curve is shown in Fig. 4b, calibration curves were generated for all four green tea catechins as EGCG is metabolized to EGC and ECG metabolized to EC [40] (GTCs as previously defined in Fig. 2). Calibration curves were reproducible and linear for each compound over the range 0.1–10 µg/ml, with *R*^2^ values >0.99 %, indicating a strong relationship between peak area and concentration (Fig. 4c).

The cream A formulation were assessed to see precisely how much of each particular catechin was present. 5 µl of cream A was dissolved in 500 µl citrate phosphate buffer (1:100 dilution) and run as a standard (in quintuplet from 5 separate runs), which contained on average 21.2 mg/ml of EGCG, with a coefficient of variation of 10.2 %. EC was detected at 0.67 mg/ml, and EGC and ECG both detected at a low level (0.08 and 0.10 mg/ml respectively).

When whole tissue biopsies treated with one dose of 5 µl cream A in the short term organ culture experiments performed as described above were analysed, EGCG was detected at low concentrations. EGCG was detected after 1 h at 0.17 µg/ml in the citrate phosphate extraction buffer, peaking at 0.29 µg/ml after 4 h, and was detectable up to 48 h after dosage. EGC, EC and ECG were not detected in the tissue, and no GTCs were detected in the culture media over 48 h.

### Histology

**Fig. 5:**
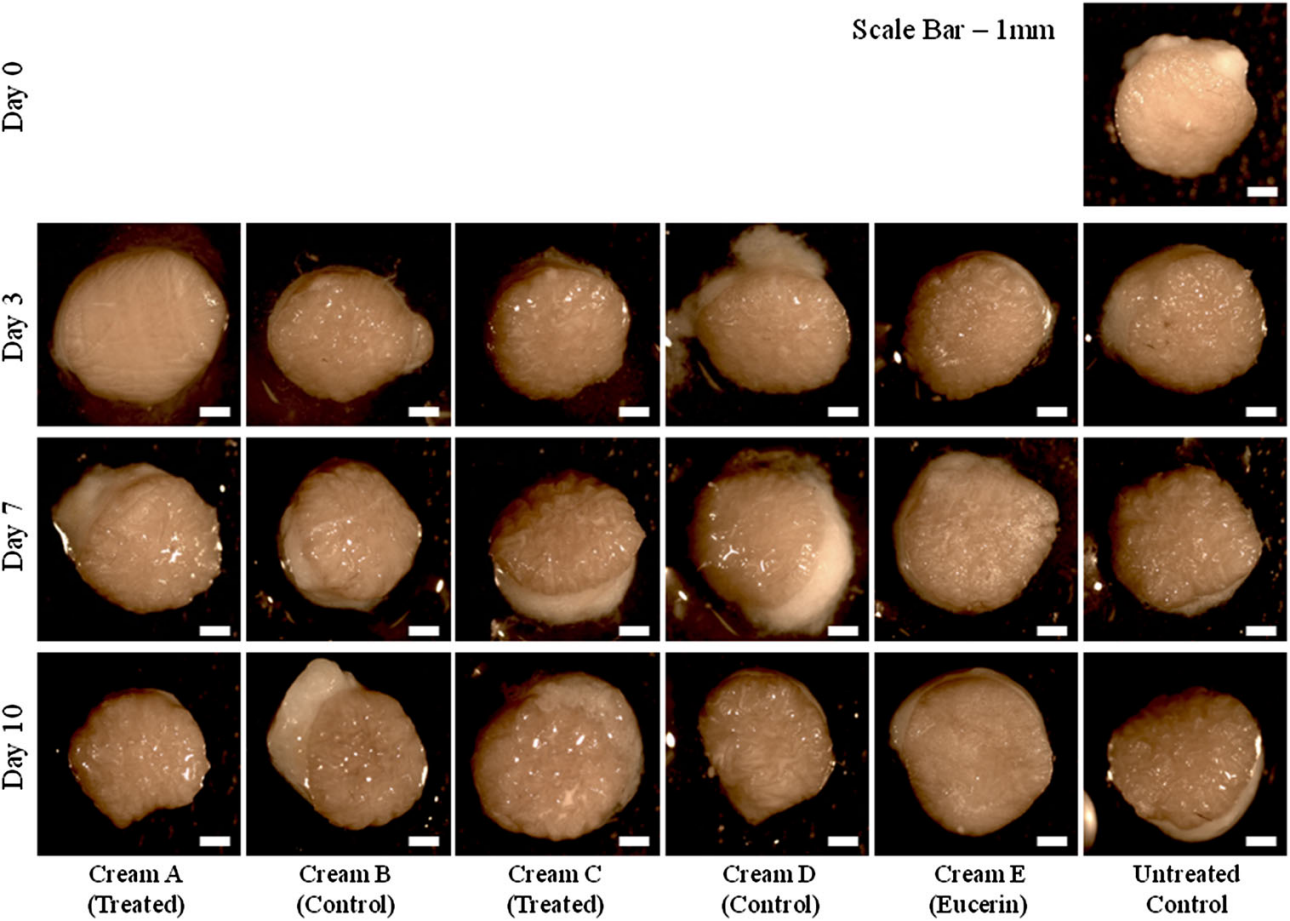
Photographs of 6 mm punch biopsies removed from whole tissue organ culture experiments after 3, 7 and 10 days, for treated samples and untreated controls. After 10 days, there was no observable difference in tissue morphology, size, integrity or skin colour between the untreated controls and the treated samples. *Scale bar* 1 mm

**Fig. 6:**
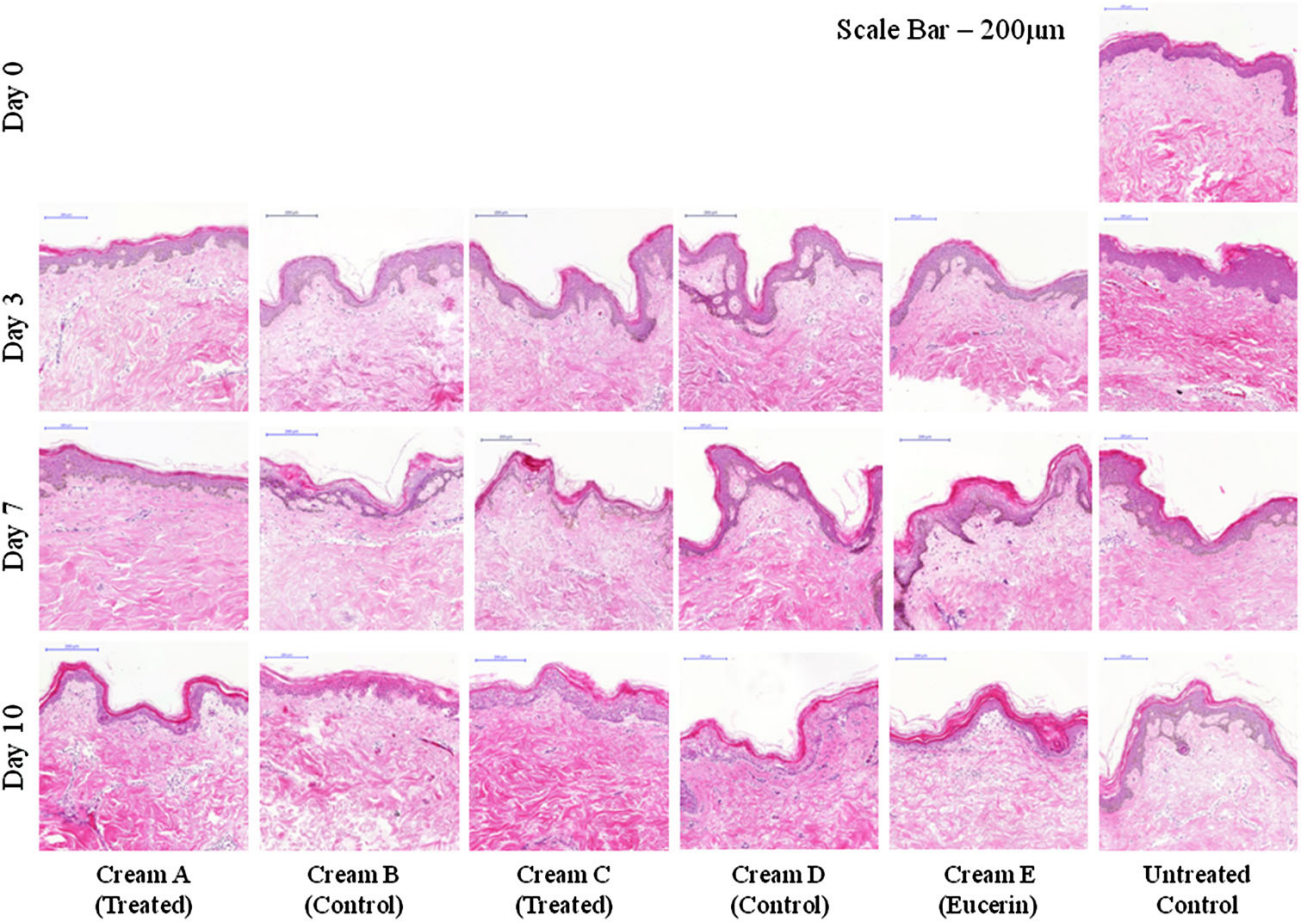
Immunohistological analysis of tissue morphology in an organ culture model using hematoxylin and eosin (*H* and *E*) staining. Day 0, Day 3, Day 7 and Day 10 untreated biopsies (no formulation used) were included as a control. There was little difference in epidermal cell layer thickness between day 0, day 3, day 7 and day 10, nor in morphology between the different green tea formulations and the treated and untreated controls. *Scale bar* = 200 µm

Tissue biopsies were removed from the whole tissue organ culture model, washed and photographed prior to processing (Fig. 5). After 10 days, there was no observable difference in tissue morphology, size, integrity or skin colour between the untreated controls and the treated samples. Tissue morphology over the 10 day culture period was further investigated using haematoxylin and eosin staining (Fig. 6). There was little difference in epidermal cell layer thickness between day 0, day 3, day 7 and day 10, however there was some swelling of the stratum corneum layer in the cultured samples over time. There was no difference in morphology between the different green tea formulations, nor between the treated and untreated controls.

**Fig. 7:**
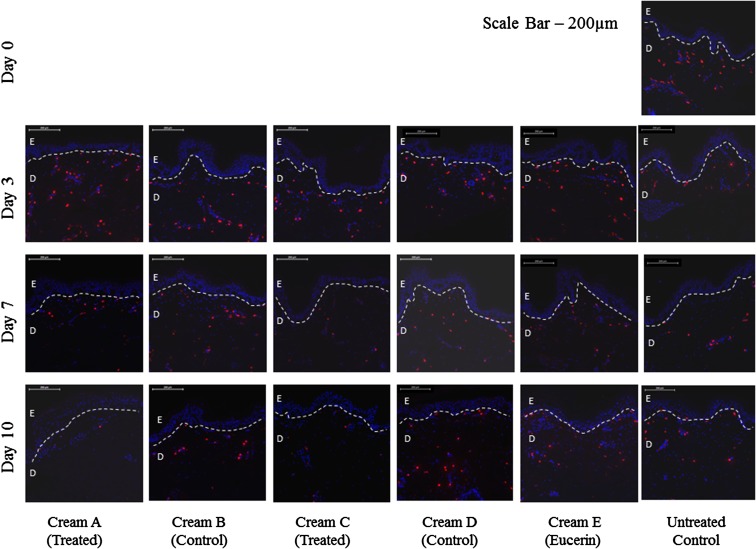
Immunohistological analysis of mast cell tryptase protein expression. Day 0, Day 3, Day 7 and Day 10 untreated biopsies (no formulation used) were included as a control. Mast cell numbers were reduced in Cream *A* and Cream *C* after 7 and 10 days, compared with treated and untreated controls. *Scale bar* = 200 µm

**Fig. 8:**
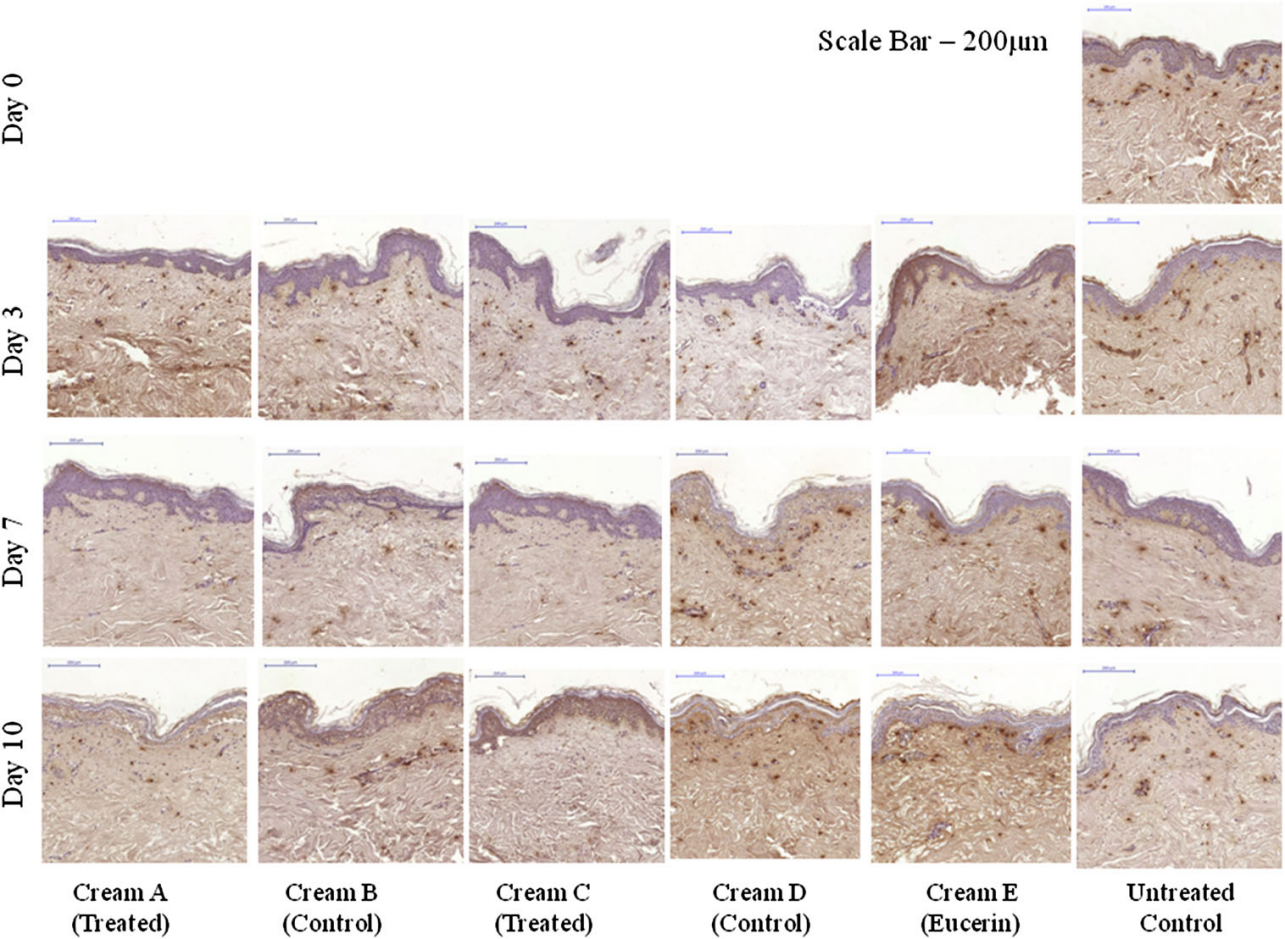
Immunohistological analysis of mast cell chymase expression in an organ culture model using peroxidise staining. Day 0, Day 3, Day 7 and Day 10 untreated biopsies untreated biopsies (no formulation used) were included as a control. Mast cell numbers were reduced in cream *A* and cream *C* after 7 and 10 days, compared with treated and untreated controls. *Scale bar* = 200 µm

Protein expression in the long term organ culture model was determined using histology, and quantified using the Definiens Tissue Studio software suite. An untreated biopsy taken at day 0 was used as a starting control, while an untreated biopsies taken from the organ culture model after 3, 7 and 10 days were also used as an internal control. Target proteins were mast cell tryptase (Fig. 7) and mast cell chymase (Fig. 8). Both mast cell tryptase and mast cell chymase were detected in the treated biopsies after 3, 7 and 10 days, and were quantified with respect to control tissues by fluorescence or peroxidise staining intensity and cell count, normalised with respect to area to give a density.

For both mast cell tryptase and mast cell chymase, the highest cell density and fluorescence density were observed at day 0. There was a reduction in cell count and fluorescence density in 3, 7 and 10 day treated and untreated controls compared with the day 0 control, however this was not significant. There was a further reduction in mast cell tryptase and mast cell chymase expression in the treated biopsies after 3, 7 and 10 days compared with the respective treated and untreated controls at the same time point (*p* < 0.05 at day 7 and 10). However there was no discernible difference between the two treatment formulations.

## Discussion

When treated samples were compared to their respective treated controls, there was reduced gene expression of α-SMA, fibronectin, mast cell tryptase, mast cell chymase, TGF-β1, CTGF and PAI-1. Histological data has shown that the tissue biopsies maintain their structure and integrity up to 10 days when treated, and that mast cell numbers, as quantified by mast cell chymase and tryptase staining, are reduced compared with both treated and untreated controls and compared with day 0 (*p* < 0.05). The differences in gene expression observed between the two cream formulations may be attributed to the additional active ingredients between the two formulations. Formulation 1 (Cream A and B) contained Hydrolysed Algin, Chlorella Vulgaris, Magnolia Officinalis Bark Extract and Vitis Vinifera Seed Extract, whereas formulation 2 (Cream C and D) contained Aloe Barbadensis Leaf Juice, Tocopherol and Sodium Hyaluronate, all of which are commonly used in cosmetics as moisturising agents (Table 1). In addition, formulation 1 contained 5 % *Camellia sinensis* extract, whereas formulation two contained 5 % EGCG. As discussed earlier in the results, EGCG is the main active catechin present in *C*. *sinensis* extract and has the highest anti-oxidant activity due to the number of hydroxyl groups present in its structure [29, 36], this is metabolized to EGC and ECG metabolized to EC [40], which guided our decision to use EGCG in the second topical formulation.

The use of ex vivo whole skin biopsies in culture allows the effect of individual ingredients and formulations to be tested [33], and a number of different approaches exist. Utilising a collagen matrix embedding protocol, skin biopsies are fixed in culture plates [1, 12]. In work previously published by our group, green tea catechins were shown to inhibit keloid tissue growth and induce biopsy shrinkage using the collagen embedding methodology, also indicating altered expression of many of the markers identified and chosen for this study [38]. In the modified approach used in this study, transwell inserts containing a fine mesh bottom are used to suspend the biopsy in culture media [27]. This has the added benefit of keeping the epidermis exposed, allowing for topical treatments to be applied.

The health benefits and anti-oxidant activity of *C*. *sinensis* have been known for centuries [29, 36], reducing the potential damaging effect of reactive oxygen species and free radicals, as well as inhibiting cell signalling pathways linked to cancer, inflammation and autoimmune disease [4, 19, 20, 25, 41, 42]. The amount of catechins present in green tea can vary dependant on batch, number of cups drank daily and brew strength, with catechins degrading when brewed due to temperature [5, 21]. Transdermal delivery avoids some of the issues associated with oral delivery, such as first pass metabolism in the liver and gastrointestinal uptake of molecules, with localized dosage targeting the skin [14, 21].

Topical applications of GTCs have been shown to have a beneficial effect on a range of pathways in different models. In an artificial skin culture model GTC decreased the level of MMPs production and increased TIMP-1 expression level, and reduced alterations in the extracellular matrix following application of UVA radiation [25]. A comparable UVA/UVB radiation study undertaken in a mouse model using EGCG and ginkgo biloba showed that the two extracts had a complimentary photoprotective effect, linked to a biological/free radical scavenging effect as opposed to a UV-filtering effect [7]. GTCs also play a role in angiogenesis. In a small scale double blind clinical study in human subjects suffering from erythema and telangiectasia, catechin containing topical treatment reduced both HIF-1 alpha and VEGF expression [10]. In a separate study in aged skin, a combination of oral green tea and topical cream increased skin elastic tissue content [6].

It has been shown that GTCs significantly inhibit mast cell stimulated type I collagen expression by suppressing activation of the PI-3k/Akt/mTOR signalling pathways in keloid fibroblast culture [43]. High concentrations of EGCG reversibly regulated the cell growth and expression of cell cycle-related proteins and genes in normal fibroblasts, inhibiting cancer cell line proliferation while leaving normal cells unaffected at an appropriate dosage [15]. EGCG suppresses keloid fibroblast pathogenesis by inhibiting STAT3 signalling, but that although EGCG inhibited PI3K and MEK/ERK signalling, did not further block proliferation, migration, or collagen production in KFs treated with STAT3 inhibitors [31].

We also demonstrated that topical cream formulations containing GTC reduces mast cell numbers in a normal skin organ culture model. This finding has implications for the treatment of a range of mast cell influenced skin disorders, such as contact dermatitis, atopic dermatitis, psoriasis and scleroderma where number of mast cells are increased [3, 17, 23, 26, 30, 39]. We have shown that GTC from different cream formulations delivered to the stratum corneum of normal human skin are retained in the skin, consistent with previously published findings. GTCs fit the profile for being suitable for transdermal delivery [37], and have previously been analysed in mouse pig and human skin models, with differing rates of uptake through the stratum corneum into the epidermis and dermis in each animal model [2, 8, 13, 24]. Further long term clinical studies in normal and diseased human skin are required in order to establish the efficacy and utility of this therapeutic approach.

In conclusion, this skin model can be utilised as a further tool alongside already established cell culture based models to analyse not only cosmaceuticals and transdermal delivery, but can also be adapted to study skin biopsies taken from a range of conditions in order to assess wound healing mechanisms, fibrosis or other skin diseases. This approach may also prove a useful tool in the future development of new cosmeceuticals and understanding disease alongside already established techniques, in order to give a further level of validation to the data produced whilst avoiding the need for using animal models.

## Abbreviations

GTE: Green tea extract
GT: Green tea catechins
ECM: Extracellular matrix
α-SMA: α-smooth muscle actin
TGF-β1: Transforming growth factor-β1
CTGF: Connective tissue growth factor
PI3-K: Phosphatidylinositide 3-kinase
PAI-1: Plasminogen activator inhibitor-1
STAT3: Signal transducer and activator of transcription 3
MMP-3: Matrix metalloproteinase-3
RPL32: Ribosomal protein L32
